# Treatment with a New Peroxisome Proliferator-Activated Receptor Gamma Agonist, Pyridinecarboxylic Acid Derivative, Increases Angiogenesis and Reduces Inflammatory Mediators in the Heart of *Trypanosoma cruzi*-Infected Mice

**DOI:** 10.3389/fimmu.2017.01738

**Published:** 2017-12-11

**Authors:** Federico Nicolás Penas, Davide Carta, Ganna Dmytrenko, Gerado A. Mirkin, Carlos Pablo Modenutti, Ágata Carolina Cevey, Maria Jimena Rada, Maria Grazia Ferlin, María Elena Sales, Nora Beatriz Goren

**Affiliations:** ^1^Departamento de Microbiología, Parasitología e Inmunología, Facultad de Medicina, Universidad de Buenos Aires, Buenos Aires, Argentina; ^2^Instituto de Investigaciones en Microbiología y Parasitología Médica (IMPaM)-CONICET, Buenos Aires, Argentina; ^3^Department of Pharmaceutical and Pharmacological Sciences, University of Padova, Padova, Italy; ^4^Centro de Estudios Farmacológicos y Botánicos (CEFyBO)-CONICET, Facultad de Medicina, Universidad de Buenos Aires, Buenos Aires, Argentina; ^5^Instituto de Química Biológica, Facultad de Ciencias Exactas y Naturales (IQUIBICEN)-CONICET, Universidad de Buenos Aires, Buenos Aires, Argentina; ^6^Universidad de Buenos Aires, Consejo Nacional de Investigaciones Científicas y Técnicas, Instituto de Investigaciones Biomédicas en Retrovirus y Sida (INBIRS), Facultad de Medicina, Buenos Aires, Argentina

**Keywords:** *Trypanosoma cruzi*, angiogenesis, new peroxisome proliferator-activated receptor gamma agonist, macrophages, inflammatory mediators, heart fibrosis

## Abstract

*Trypanosoma cruzi* infection induces an intense inflammatory response in diverse host tissues. The immune response and the microvascular abnormalities associated with infection are crucial aspects in the generation of heart damage in Chagas disease. Upon parasite uptake, macrophages, which are involved in the clearance of infection, increase inflammatory mediators, leading to parasite killing. The exacerbation of the inflammatory response may lead to tissue damage. Peroxisome proliferator-activated receptor gamma (PPARγ) is a ligand-dependent nuclear transcription factor that exerts important anti-inflammatory effects and is involved in improving endothelial functions and proangiogenic capacities. In this study, we evaluated the intermolecular interaction between PPARγ and a new synthetic PPARγ ligand, HP_24_, using virtual docking. Also, we showed that early treatment with HP_24_, decreases the expression of NOS2, a pro-inflammatory mediator, and stimulates proangiogenic mediators (vascular endothelial growth factor A, CD31, and Arginase I) both in macrophages and in the heart of *T. cruzi*-infected mice. Moreover, HP_24_ reduces the inflammatory response, cardiac fibrosis and the levels of inflammatory cytokines (TNF-α, interleukin 6) released by macrophages of *T. cruzi*-infected mice. We consider that PPARγ agonists might be useful as coadjuvants of the antiparasitic treatment of Chagas disease, to delay, reverse, or preclude the onset of heart damage.

## Introduction

Chagas disease (American trypanosomiasis) is caused by the protozoan parasite *Trypanosoma cruzi*. The acute phase of infection is characterized by the presence of parasites in the host bloodstream and diverse tissues.

Acute *T. cruzi* infection is characterized by parasite invasion of the heart and other organs. Monocytes that are recruited from blood to the heart differentiate into macrophages that mediate the control of the parasite load. Macrophages produce pro-inflammatory mediators *in situ*, such as nitric oxide (NO), TNF-α, and interleukin 6 (IL-6), that inhibit *T. cruzi* multiplication and differentiation, precluding the spread of the infection within the host (1). In this regard, some studies have shown that mice administered iNOS inhibitors exhibit higher parasite levels and greater mortality than untreated mice (2, 3). Also, other authors have shown the relevant role of TNF-α in protection of mice during the acute *T. cruzi* infection (4–6).

On the other hand, a pro-inflammatory response may precipitate pathological conditions. In Chagas disease, the antigenic stimuli persist for years. The expression of pro-inflammatory mediators such as tumor necrosis factor-alpha (TNF-α), IL-6, interleukin 1 beta (IL-1β), and nitric oxide synthase 2 (NOS2) is associated with progressive tissue damage, leading to cardiac pathological conditions (7–11).

The infection-associated immunopathology and microvascular abnormalities are crucial aspects in the generation of heart disease, which is characterized by myocytolysis, thromboembolism, dysrhythmia, and cardiac hypertrophy (12). The progression of the disease is favored by these features, which lead to a gradual reduction in coronary flow. In this microenvironment M2 macrophages might play a crucial role, because they contribute to a range of physiological processes, including homeostasis, repair, metabolic functions, and angiogenesis, by secreting a plethora of proangiogenic factors like vascular endothelial growth factor A (VEGF-A), CD31, NOS3, and other cytokines, which increase the proliferation of endothelial and epithelial cells to induce neovascularization (13–15).

Peroxisome proliferator-activated receptor gamma (PPARγ) is a ligand-dependent transcription factor of the nuclear receptor superfamily, involved in the regulation of lipid metabolism, insulin sensitivity, and inflammatory response (16). It has been suggested that PPARγ is involved in the molecular mechanisms that regulate neoangiogenesis, through the action of growth factors and cytokines that stimulate migration, proliferation, and survival of endothelial cells (17). PPARγ ligands enhance VEGF-A expression in human vascular smooth muscle cells and upregulate NOS3 expression in myocardial infarction (18, 19). Particularly, it has been demonstrated that troglitazone enhances the expression of VEGFR-2 in HUVEC cells through PPARγ activation (20). However, some studies have shown that PPARγ and PPARα activation inhibits angiogenesis *in vitro* and *in vivo*, affecting vascular remodeling and leading to reduction of tumor cell growth (21). Thus, it is important to undertake studies to increase the knowledge about the possible role of PPARγ receptor and its ligands in heart angiogenesis, particularly in Chagas disease.

The new PPARγ synthetic ligand 3-hydroxy-4-pyridinecarboxylic acid derivative 24 (HP_24_), an aza-analog of salicylic acid and structurally close to other potent anti-inflammatory pyridine compounds, has been tested in dextran sulfate sodium-induced colitis in mice, where it showed a significant decrease in colonic myeloperoxidase activity and IL-1β tissue levels, exhibiting its anti-inflammatory activity without cytotoxic activity (22).

Pharmacological interventions leading to enhanced vascular development and reduction of inflammation and fibrosis might be useful to prevent heart functional abnormalities. The aim of this study was to determine the effect of the new PPARγ ligand HP_24_ in angiogenesis and in the levels of inflammatory mediators and to analyze the participation of macrophages in these processes.

## Materials and Methods

### Ethics Statement

The BALB/c mice used in this study were bred and maintained in the animal facility at the Instituto de Investigaciones en Microbiología y Parasitología Médica (IMPaM), Universidad de Buenos Aires–CONICET. All the procedures were approved by the Institutional Committee for the Care and Use of Laboratory Animals (CICUAL, Facultad de Medicina, Universidad de Buenos Aires, CD No. 2271/2014) and are in accordance with the guidelines of the Argentinean National Administration of Medicines, Food and Medical Technology (ANMAT), Argentinean National Service of Sanity and Agrifoods Quality (SENASA) and also based on the US NIH Guide for the Care and Use of Laboratory Animals.

### Mice and Infection

All mice were provided with a 12-h day/night cycle and water and food *ad libitum* with a standard diet. Seven male mice per group were infected intraperitoneally with 1 × 10^5^ bloodstream trypomastigotes of a lethal RA (pantropic/reticulotropic) subpopulation of *T. cruzi* (23) and sacrificed by CO_2_ inhalation at 10 days postinfection (dpi). Each experiment was carried out at least three times.

### Synthesis of 1-Methyl-3-Hydroxy-4-Pyridinecarboxylic Acid Derivative 24 (HP_24_)

**Figure 1:**
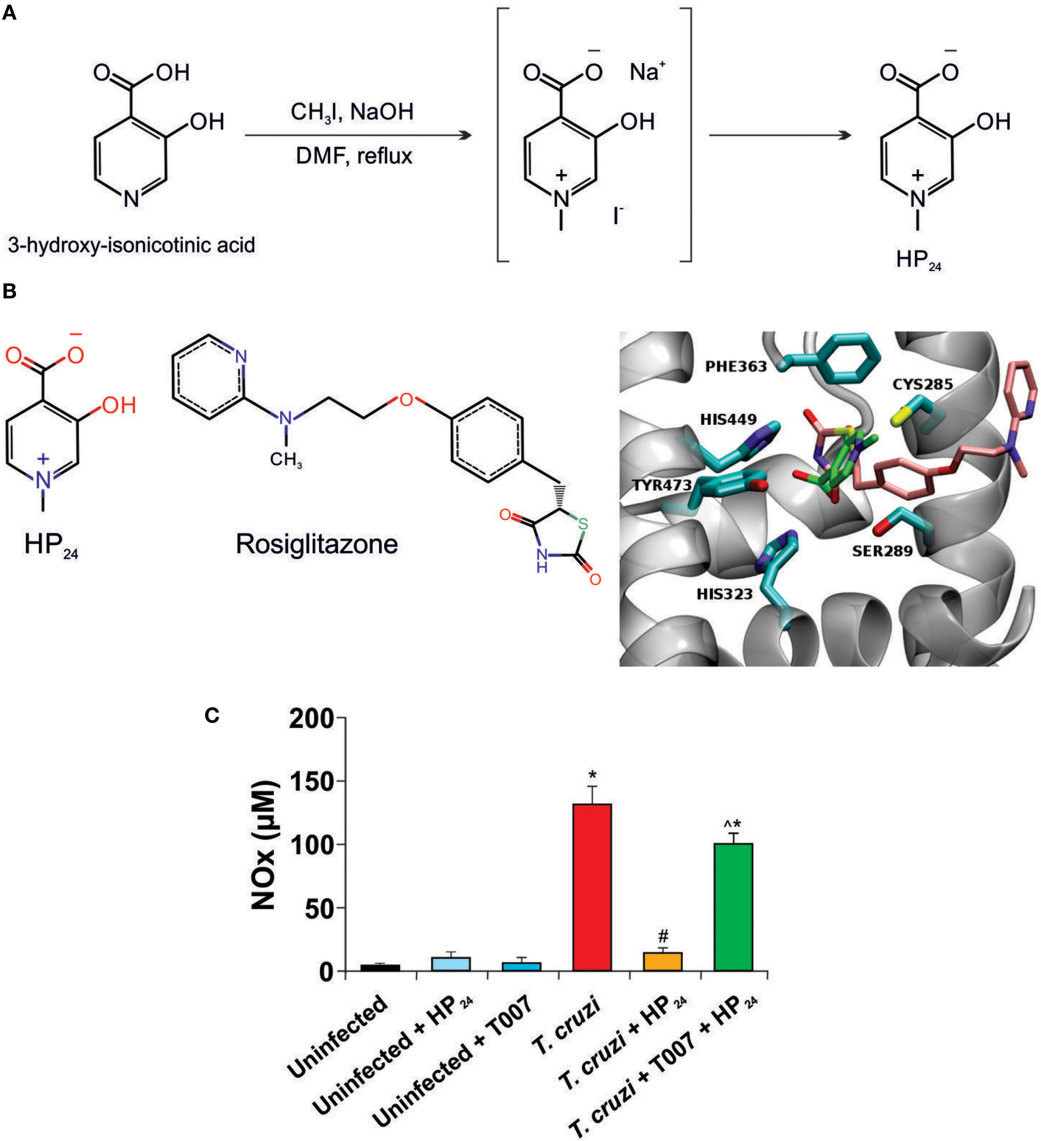
HP_24_ interacts with peroxisome proliferator-activated receptor gamma (PPARγ). **(A)** Route for HP_24_ synthesis. **(B)** Predicted binding mode for HP_24_ to PPARγ. On the right panel, we show rosiglitazone (RSG) from complex crystallographic structure (PDB id 2PRG) superimposed to most probably conformation of HP_24_ compound from docking calculations. RSG, HP_24_, and receptor binding site amino acids are shown in stick representation, colored by atom type (oxygen, nitrogen, sulfur in red, blue, and yellow, respectively) except carbons, which are colored in pink, green, and cyan respectively. Receptor backbone is shown in gray with New Ribbon representation. On the left panel, we show chemical formula of RSG and HP_24_. The images were generated using VMD software (24). **(C)** Peritoneal macrophages from *Trypanosoma cruzi*-infected mice were preincubated with T0070907 (50 µM), a PPARγ specific antagonist, for 30 min. Then, incubated with HP_24_ (100 µM). After 48 h, nitrite accumulation was measured in the cultured medium. For panel **(C)**, results represent the mean ± SEM of three independent experiments (five mice per group). *P < 0.05 vs. uninfected cells, ^#^*P* < 0.05 vs. *T. cruzi*-infected cells. ^^^P < 0.05 vs. *T. cruzi-*infected HP_24_-treated cells.

1-Methyl-3-hydroxy-4-pyridinecarboxylic acid derivative was resynthesized following the previously reported pathway by Brun et al. (22) with some modifications in the reaction conditions for the final steps of the synthesis and the purification step that led to the desired compound HP_24_ in the zwitterion form (Figure 1A) instead of the previously described chloride compound. 3-Hydroxy-isonicotinic acid (1 g, 7.18 mmol) was suspended in 5 ml of DMF in a 25-ml round bottomed flask. The resulting suspension was stirred at room temperature, and 10% NaOH (7.5 ml) was added dropwise until complete dissolution of the solid (pH 9–10). Methyl iodide (2.06 g, 14.46 mmol, *d* = 2.28 g/ml, 0.9 ml) was added under stirring and the solution was then refluxed, monitoring the reaction progress by thin layer chromatography (*n*-butanol:H_2_O:AcOH, 1:1:1). Once the starting material disappeared, the solvent was removed under reduced pressure, obtaining a deep orange colored solid, which was dissolved in boiling water (50 ml). The solution was acidified with 37% HCl (3.5 ml), and 10% H_2_O_2_ (1 ml) was added. Then, the iodine was exhaustively extracted with CHCl_3_ (5× 15 ml) in a separating funnel. The organic phase was concentrated under pressure to dryness, obtaining an orange crude raw powdery solid (1.662 g), which was purified by reversed-phase chromatography in a Biotage Isolera Spektra Flash Chromatography apparatus equipped with prepacked C18 cartridges. The fractions containing the product were pooled and concentrated to dryness by means of a rotary evaporator, yielding a white powdery product (0.956 g, 6.21 mmol).

#### 3-Hydroxy-1-Methylpyridin-1-Ium-4-Carboxylate (HP24)

Yield: 86.4%; mp: 236°C (decomposition); *R*_f_: 0.13 (*n*-butanol:H_2_O:AcOH, 1:1:1); IR (KBr): ν (cm^−1^) = 3,432 (OH), 3,079 (=C–H), 2,850 (CH_3_), 1,654 (COO^−^), 1,480 (C=C), 1,381 (C=N), 1,300 (C–N) cm^−1^; ^1^H NMR (300 MHz, [D6] DMSO) δ = 8.43 (s, 1H, H-2), 8.01 (d, *J* = 6.00 Hz, 1H, H-6), 7.98 (d, *J* = 6.03 Hz, 1H, H-5), 4.19 ppm (s, 3H, N-CH_3_); ^13^C NMR (75 MHz, [D6] DMSO) δ = 47.98 (N-CH_3_), 126.85 (C-5), 129.73 (C-4), 130.72 (C-6), 137.54 (C-2), 164.99 (C-3), 166.95 ppm (COO^−^); HRMS (ESI-MS, 140 eV): *m*/*z* [M + H^+^] calculated for ${\text{C}}_{7}{\text{H}}_{8}{\text{NO}}_{3}^{+}$, 154.0504; found, 154.0545; RT-HPLC, C18: *t*_R_ = 5.40 min, 97.61 A%; elemental analyses: calculated for C_7_H_7_NO_3_, C 54.9%, H 4.61%, N 9.15%; found: C 54.47%, H 4.39%, N 8.98%.

### Treatment of Mice with HP_24_

Mice were treated by oral gavage with HP_24_ (400 mg/kg/day) suspended in phosphate-buffered saline (PBS), according to Brun et al. (25), for 9 days, since day 1 postinfection (pi), and sacrificed at day 10 pi. Euthanasia was carried out at this time for humanitarian reasons due to the unhealthy conditions of the mice infected with the RA parasite strain.

### Parasitemia, Body Weight, and Survival of Mice

Parasitemia of *T. cruzi*-infected mice was analyzed through a small incision at the end of the tail. Blood from *T. cruzi*-infected mice and *T. cruzi*-infected HP_24_-treated mice was obtained at 3, 5, 8, and 10 dpi. Fivefold dilutions were obtained in red blood cell lysis buffer (150 mM NH_4_Cl, 0.1 mM EDTA, and 10 mM KHCO_3_, pH 7.4). Parasitemia was measured in a Neubauer chamber. Body weight gain or loss was monitored on the days described previously. For survival studies, two independent groups (*T. cruzi*-infected and *T. cruzi*-infected HP_24_-treated mice) of seven animals each were followed up daily until different days postinfection and analyzed by the Kaplan–Meyer method.

### Molecular Modeling

To elucidate the binding mode of the HP_24_ molecule, docking calculations were performed against a murine PPARγ receptor. First, we built an homology model with MODELLER software (26) using human PPARγ structures as template from Protein Data Bank (http://www.pdb.org), PDB id 5DV6 and 2PRG (27). Then, we built and optimized HP_24_ ligand structure with Molefacture module of VMD software (24) employing default settings for convergence criteria. Finally, AutoDock4 software (28) with Water Site Biased Method (29) was used for docking experiments. The PPARγ binding site (BS) was defined as all atoms within a radius of 10 Å from the co-crystallized ligand rosiglitazone (RSG, PDB id 2PRG). The receptor BS amino acids were treated as rigid structures during the calculations. A total of 100 different docking runs were performed, and the results were clustered according to the ligand-heavy atom RMSD using a cutoff of 2 Å. The genetic algorithm parameters for each conformational search run were kept at their default values. The AutoDock4.2 energy function (OA atom type map) was modified adding an additional energy term for each crystallographic water (CWS) placed on ABS BS in the apo form (PDB id 1PRG) to the original function (29).

### Isolation of Peritoneal Macrophages

Macrophages were obtained by washing the peritoneal cavity of mice with 8 ml of RPMI-1640 culture medium (Invitrogen Life Technologies, Grand Island, NY, USA), supplemented with 10% of heat-inactivated fetal bovine serum (FBS) (Internegocios S.A., Argentina) and antibiotics (50 µg/ml of penicillin, streptomycin, and gentamicin). Cells were left to adhere to the plastic surface of cell culture dishes, 35 mm × 10 mm (Greiner Bio One International AG) for 3 h at 37°C under a 5% CO_2_ atmosphere (30).

### Treatment with HP_24_ and T0070907 *In Vitro*

Peritoneal macrophages were isolated from *T. cruzi*-infected mice as indicated above. The cells were preincubated for 30 min with specific PPARγ antagonist, T0070907 (50 µM in DMSO) (Sigma-Aldrich Co., St. Louis, MO, USA), and treated afterward with HP_24_ (100 µM in PBS) for 48 h. Cell viability was examined by Trypan Blue dye exclusion test, for each treatment.

### Measurement of NO Production by Peritoneal Macrophages *In Vitro*

Release of NO by peritoneal macrophages from *T. cruzi-*infected mice was assessed by the Griess reaction, as previously described (31). The absorbance at 540 nm was compared with a standard curve of NaNO_2_.

### Macrophage-Induced Angiogenesis

Macrophage-induced angiogenesis was quantified with an *in vivo* bioassay. After detaching, the concentration of macrophages was adjusted to 2 × 10^5^ cells/ml in culture medium without FBS. Seven normal syngeneic male mice *per* group were inoculated intradermally in both flanks with 0.1 ml of cell suspension. Five days after inoculation, mice were sacrificed, and the internal layer of skin was separated from the underlying tissues, and the vascular response was observed with a dissecting microscope (Konus USA Corporation, Miami, FL, USA) at a 7.5× magnification and photographed with an incorporated digital camera (Canon Power Shot A45, Canon USA, Inc., Lake Success, NY, USA). Photos were projected on a reticular screen to count the number of vessels per square millimeters of skin. Angiogenesis was quantified as vessel density, calculated as the total number of vessels divided by the total number of squares (32).

### Coculture of Macrophages with Heart Explants

Macrophages (1.5 × 10^6^) from uninfected, *T. cruzi*-infected and *T. cruzi*-infected HP_24_-treated mice were obtained and cultured with heart slices (100 mg/sample) from uninfected and *T. cruzi*-infected mice in 4 ml RPMI-1640 culture medium (Invitrogen Life Technologies, Grand Island, NY, USA). After 48 h, the culture supernatants were collected, and hearts were homogenized to obtain total proteins as previously described (33).

### Histopathological Studies

Hearts of all experimental groups were fixed in 4% paraformaldehyde in PBS, dehydrated and embedded in paraffin. Six non-contiguous sections (5 µm) were stained with hematoxylin–eosin or Masson trichrome stain. Cellular infiltrates, presence of amastigote nests and collagen deposition were examined in using a Nikon Eclipse E600 microscope (Nikon Inc.). Images were captured with a Spot RT digital camera. At least 30 random microscopic fields (400×) were analyzed in each microscopic section, using the open source Image J software (NIH, USA) (34).

### Quantitative Real-time Reverse-Transcriptase Polymerase Chain Reaction (RT-qPCR)

Total RNA was extracted from frozen cells by using a QuickZol reagent (Kalium Technologies, Buenos Aires-Argentina). Total RNA was reverse-transcribed using Expand Reverse Transcriptase (Invitrogen Corp., MA, USA). RT-qPCR was performed using a 5× HOT FIREPol^®^ EvaGreen^®^ qPCR Mix Plus (ROX) (Solis BioDyneCorp., Estonia) in an Applied Biosystems 7,500 sequence detector. Primer sequences were: 18S: Fw 5′AACACGGGAAACCTCACCC 3′, Rv 5′ CCACCAACTAAGAACGGCCA 3′; connective tissue growth factor (CTGF): Fw 5′ CCTAAAATCGCCAAGCCTGT 3′, Rv 5′ CACCCCGCAGAACTTAGCC 3′, and PPARγ: Fw 5′ ATCTACACGATGCTGGC 3′, Rv 5′ GGATGTCCTCGATGGG 3′; PCR parameters were 52°C for 2 min, 95°C for 15 min, and 40 cycles of 95°C for 30 s and 60°C (for 18S), 63°C (for CTGF) or 54°C (for PPARγ). Quantification was calculated using the comparative threshold cycle (*C*_t_) method and the efficiency of the RT reaction $(relative\;quantity,2^{-\Delta \Delta C_{t}}).$ The replicates were then averaged, and fold induction was determined, considering the value at time 0 as 1 (35).

### Determination of Cytokine Levels

TNF-α and IL-6 levels in culture supernatants were quantified by enzyme-linked immunosorbent assays using DuoSet antibody pairs (R&D Systems, Minneapolis, MN, USA).

### Preparation of Total Protein Extracts for Western Blot

Total protein extracts were obtained after washing the hearts with PBS and adding 300 ml of RIPA modified lysis buffer (50 mM NaCl, 50 mM Tris–HCl (pH 7.40), 1% Triton X-100, 1 mM EDTA, 1 mM PMSF; 2.5 g/l Protease Inhibitor Cocktail (Sigma-Aldrich Co., St. Louis, MO, USA), 1 mM Na_3_VO_4_, 1 mM NaF), or washing the cultured cells and scraped off the dishes with 50 µl of the same buffer. Then, the tubes were kept on ice for 30 min with swirling, and the samples were centrifuged at 7,000 *g* at 4°C for 10 min. The supernatants were stored at −20°C. Protein concentrations were determined by the Bradford method using the Bio-Rad Protein Assay (Bio-Rad, USA) and bovine serum albumin (Sigma-Aldrich Co., St. Louis, MO, USA) as a standard (36). For Western blot analysis, total proteins were boiled in Laemmli sample buffer, and equal amounts of protein (40–50 µg) were separated by 10–12% SDS-PAGE. The gels were blotted onto a Hybond-P membrane (GE Healthcare, Madrid, Spain) and incubated with the following antibodies: anti-NOS2, anti-NOS3, anti-Arginase I (Arg-I), anti-CD31, anti-VEGF-A, and anti-α-actin (Santa Cruz Biotechnology, CA, USA). The blots were revealed by enhanced chemiluminescence in an Image Quant 300 cabinet (GE Healthcare Biosciences, PA, USA) following the manufacturer’s instructions. Band intensity was analyzed using the NIH-ImageJ software (37).

### Statistical Analysis

Data are expressed as the mean of three independent experiments ± SEM for each treatment (seven mice/group). The Kaplan–Meier test was used to compare survival curves between groups. One-way ANOVA was used to analyze the statistical significance of the differences observed between the uninfected, uninfected HP_24_-treated, *T. cruzi*-infected, and *T. cruzi*-infected HP_24_-treated mice. The Tukey *post hoc* test was performed to compare all pairs of groups. Kruskal–Wallis test and Dunn’s *post hoc* test was used to analyze the differences in collagen deposition between uninfected, *T. cruzi*-infected, and *T. cruzi*-infected HP_24_-treated mice. Differences were considered statistically significant when *P* < 0.05. All analyses were performed using the Prism 5.01 software (GraphPad, USA).

## Results

### The New Pyridinecarboxylic Acid Derivative 24 (HP_24_) Interacts with PPARγ

Selection of the 3,4-pyridinecarboxylic derivative HP_24_ was based on its properties as PPARγ ligand and on its ability to reduce the pro-inflammatory response in a Dextran-induced colitis mouse model, as previously reported (22). A simplified synthesis route is shown in Figure 1A.

To elucidate how HP_24_ interacts with PPARγ, we compared its binding against that of co-crystallized structure of synthetic PPARγ ligand, rosiglitazone (RSG). Figure 1B illustrates the superimposition of the best energy docking result for HP_24_ (estimated free energy of binding −4.05 kcal/mol), as well as the experimental position of the co-crystallized RSG (estimated free energy of binding −8.95 kcal/mol), present in the 2PRG PDB structure. The most important interaction between RSG and PPARγ is a hydrogen bond network with residues GLN286, SER289, HIS323, and TYR473. Hydrophobic contacts with LEU330, ILE341, MET364, and CYS285 are considered as secondary interactions (27). Besides, a detailed visual inspection of the binding modes for HP_24_ reveals the same hydrogen bond network, adding an extra-polar contact with HIS449 in the hydrophilic region of PPARγ BS (Figure 1B, panel right).

Since ligand binding can be inhibited by specific antagonists, we designed an *in vitro* assay to confirm that HP_24_ binds to PPARγ as modeled by the *in silico* binding analysis. This involved the use of NO release by *T. cruzi-*infected peritoneal macrophages, as an indicator system of the inhibitory effect of HP_24_ on pro-inflammatory mediators release. *T. cruzi-*infected macrophages release significantly higher amounts of NO than uninfected macrophages. Moreover, HP_24_ significantly reduced the release of this pro-inflammatory mediator. Notably, preincubation of *T. cruzi-*infected macrophages with the specific PPARγ antagonist, T0070907 impeded the effect of HP_24_ on NO release, thus confirming the specific binding and activation of PPARγ by HP_24_ (Figure 1C).

**Figure 2:**
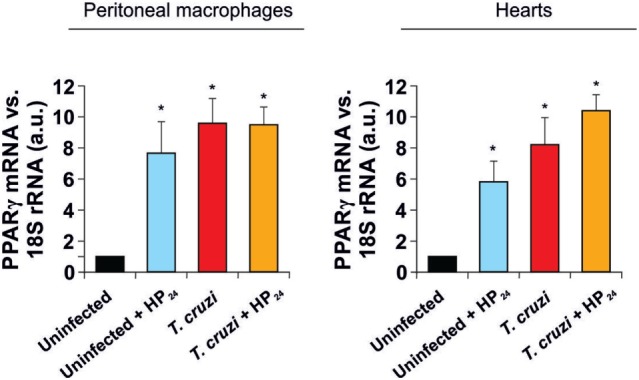
Peroxisome proliferator-activated receptor gamma (PPARγ) expression. PPARγ mRNA expression was analyzed by quantitative real-time reverse-transcriptase polymerase chain reaction in peritoneal macrophages and heart homogenates from uninfected and *Trypanosoma cruzi*-infected mice, treated or not with HP_24_. Results were normalized against 18S rRNA. Results represent the mean ± SEM of three independent experiments (five mice per group). *P < 0.05 vs. uninfected mice.

As previously reported by our group, infection of mice with *T. cruzi* increases PPARγ expression in the heart and in peritoneal macrophages (30, 38). In this study, we tested the effect of HP_24_ treatment on PPARγ mRNA levels in macrophages and hearts of uninfected and *T. cruzi*-infected mice. Figure 2 shows that HP_24_ treatment increased the PPARγ mRNA expression in uninfected mice, but did not modify the already increased expression in *T. cruzi-*infected mice.

### HP_24_ Treatment Does Not Affect the Course of Infection

We evaluated whether the treatment with HP_24_ modified the parasitemia levels, weight and survival of *T. cruzi-*infected mice. These parameters were evaluated at 3, 5, 8, 10, and 13 dpi. We found no differences in parasitemia levels, weight or survival between *T. cruzi-*infected and *T. cruzi*-infected HP_24_-treated mice (Table 1). These results are consistent with those previously reported by our group with other PPARγ ligands (34).

**Table 1:** Effect of HP_24_ on the course of infection.

	Parasitemia (× 10^6^)				Body weight (g)				Survival (%)				
		
Day	3	5	8	10	3	5	8	10	3	5	8	10	13
*Trypanosoma cruzi*	–	–	1.4 ± 0.32	2.2 ± 0.45	19 ± 0.34	22 ± 0.23	22 ± 0.31	23 ± 0.19	100	100	100	70	10
*T. cruzi* + HP_24_	–	–	1.3 ± 0.6	2.1 ± 0.27	21 ± 0.5	24 ± 0.48	22 + 0.27	25 ± 0.46	100	100	100	80	15

*BALB/c mice were infected by i.p. route with 1 × 10^5^ trypomastigotes of the lethal RA *Trypanosoma cruzi* strain and treated daily with 400 mg HP_24_/kg/day. Body weight and parasitemia were recorded daily up to day 10 postinfection (p.i.) in each experimental group. Survival was observed daily, up to the end of the experiment (day 13 p.i.) and analyzed by the Kaplan–Meyer method. The low number of surviving animals at the end of the study precluded statistical analysis of body weight and parasitemia at this time point. Results are expressed as mean ± SEM of three independent experiments (seven mice per group)*.

### Effect of HP_24_ on *T. cruzi*-Infected Macrophages

We have previously demonstrated that PPARα and PPARγ ligands promote the polarization of macrophages isolated from *T. cruzi*-infected mice toward an M2 profile (30).

**Figure 3:**
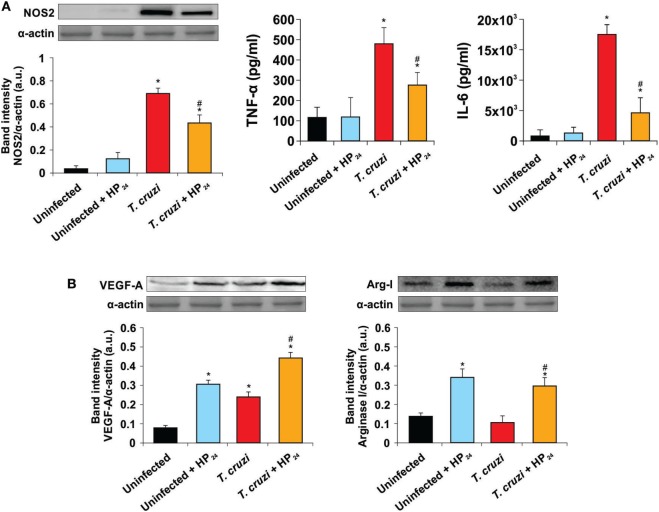
Effect of HP_24_ on *Trypanosoma cruzi*-infected macrophages. **(A)** Nitric oxide synthase 2 (NOS2) expression was determined by Western blot with a specific antibody. The release of TNF-α and interleukin 6 (IL-6) to the culture medium was quantitated by capture enzyme-linked immunosorbent assays. **(B)** Peritoneal macrophages were obtained from uninfected, uninfected HP_24_-treated, *T. cruzi*-infected, and *T. cruzi*-infected HP_24_-treated mice at 10 dpi. Vascular endothelial growth factor A (VEGF-A) and Arginase I expressions were determined by Western blot with a specific antibody. Protein levels were normalized against α-actin. Results represent the mean ± SEM band intensity of three independent experiments (five mice per group). *P < 0.05 vs. uninfected mice, ^#^*P* < 0.05 vs. *T. cruzi*-infected mice.

Here, we evaluated the role of the new PPARγ ligand HP_24_ as an anti-inflammatory ligand. We determined its effect on the expression of NOS2 and pro-inflammatory cytokines. The treatment with HP_24_ significantly inhibited NOS2 expression as well as TNF-α and IL-6 secretion in macrophages from *T. cruzi*-infected mice (Figure 3A). The PPARγ agonist did not affect the secretion of pro-inflammatory cytokines in macrophages from uninfected mice (Figure 3A).

We also analyzed whether HP_24_ was able to promote the participation of macrophages in tissue repair and neovascularization processes. To this end, we analyzed the expression of representative proangiogenic markers like VEGF-A and Arg-I in peritoneal macrophages of *T. cruzi*-infected mice. Both *T. cruzi* infection and HP_24_ treatment significantly increased VEGF-A expression in peritoneal macrophages with respect to uninfected and untreated mice. This effect was potentiated in macrophages from *T. cruzi*-infected HP_24_-treated mice (Figure 3B). However, HP_24_ was able to significantly increase the expression of Arg-I, both in *T. cruzi*-infected macrophages and in uninfected control cells (Figure 3B).

### Participation of HP_24_ in Macrophage-Induced Angiogenesis

**Figure 4:**
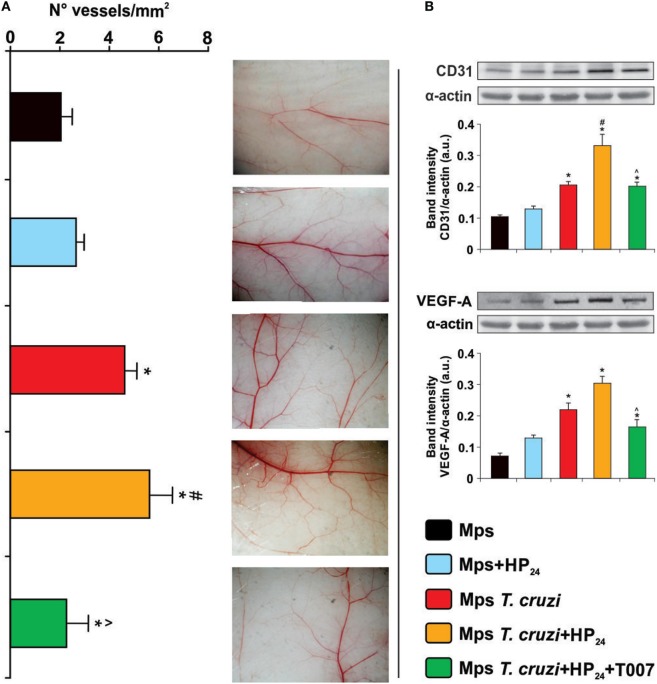
Participation of HP_24_ in macrophage-induced angiogenesis. **(A)** Macrophage-induced angiogenesis in mice was determined *in vivo* by quantification of newly formed vessels in the skin. Peritoneal macrophages from different groups were pretreated with the peroxisome proliferator-activated receptor gamma antagonist T0070907 and treated with HP_24_. Then, the cells were harvested and adjusted to 2 × 10^5^ cells/ml. One-tenth milliliters of the cell suspension was inoculated by intradermal route in both flanks of syngeneic normal mice. Five days after inoculation, recipient mice were sacrificed, the inner layer of the skin exposed, and photographs were acquired. Images were projected on a reticular screen to count the number of vessels per square millimeters. Angiogenesis was quantified as the vessel density, calculated as the total number of vessels divided by the total number of squares. **(B)** CD31 and Vascular endothelial growth factor A (VEGF-A) expression were determined by Western blot with a specific antibody. Anti-CD31 and anti-VEGF-A-specific antibody were used. Protein levels were normalized against α-actin. Results are mean band intensity ± SEM for three independent experiments (five mice per group). *P < 0.05 vs. uninfected mice macrophages, ^#^*P* < 0.05 vs. *Trypanosoma cruzi*-infected mice macrophages. ^^^*P* < 0.05 vs. HP_24_-treated macrophages from *T. cruzi*-infected mice. Mps, macrophages.

The formation of new blood vessels requires the sprouting of preexisting ones and their subsequent fusion with others (39). It has been previously reported that peritoneal macrophages from tumor-bearing mice are able to induce a strong neovascular response in the skin of syngeneic normal mice (32). Based on these results, we investigated whether HP_24_ treatment modulated the ability of peritoneal macrophages to induce neovascularization in the skin of normal syngeneic mice. Peritoneal macrophages from *T. cruzi-*infected donor mice increased vessel density in comparison with macrophages from uninfected donors. Furthermore, macrophages from *T. cruzi*-infected mice-treated *in vitro* with HP_24_ induced a higher increase in neovascularization than macrophages from *T. cruzi-*infected donor mice, upon passive transfer in the skin of normal syngeneic mice (Figure 4A). To assess the participation of PPARγ in the effects of macrophage-induced neovascularization, the cells were pretreated with the PPARγ antagonist T0070907 and then treated with HP_24_. As shown in Figure 4A the PPARγ antagonist reduced the effect of HP_24_ on new vessel formation in the skin of syngeneic normal mice.

Blood vessel density positively correlated with CD31 and VEGF-A expression. Western blot analysis showed higher expression of CD31 and VEGF-A in skin extracts of normal syngeneic recipient mice, upon transfer of macrophages from *T. cruzi-*infected mice treated *in vitro* with HP_24_, than upon transfer of macrophages from *T. cruzi*-infected mice. The PPARγ antagonist T0070907 significantly reduced the effects of HP_24_ on the expression of CD31 and VEGF-A in the skin of normal syngeneic recipient mice upon passive transfer of macrophages from *T. cruzi-*infected mice (Figure 4B).

As expected, CD31 and VEGF-A expression was higher in the skin of recipient mice transferred with macrophages from *T. cruzi-*infected mice than in mice transferred with macrophages from uninfected controls (Figure 4B).

### Effect of Macrophages from *T. cruzi*-Infected HP_24_-Treated Mice on the Expression of Proangiogenic Proteins in Heart Explants

**Figure 5:**
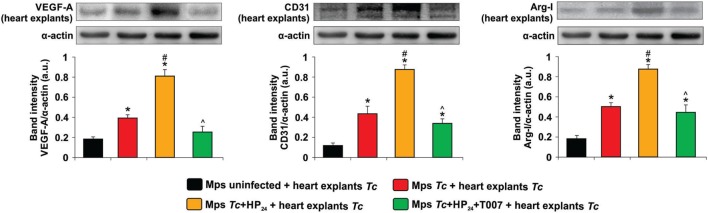
Effect of *Trypanosoma cruzi*-infected macrophages from untreated or HP_24_-treated mice on the expression of proangiogenic proteins in *T. cruzi*-infected heart explants. Peritoneal macrophages were obtained from uninfected and *T. cruzi*-infected mice at 10 dpi. Macrophages were either pretreated or not with T0070907 and then treated with HP_24_. Then, the cells were cocultured with heart explants (100 mg/sample) from *T. cruzi*-infected mice. After 48 h, heart explants were collected and homogenized. Vascular endothelial growth factor A (VEGF-A), CD31, and Arginase I (Arg-I) expressions were determined by Western blot with specific antibodies. Protein levels were normalized against α-actin. Results represent the mean band intensity ± SEM of three independent experiments (five mice per group). *P < 0.05 vs. macrophages from uninfected mice + heart explants from *T. cruzi*-infected mice, ^#^*P* < 0.05 vs. macrophages from *T. cruzi*-infected mice + heart explants from *T. cruzi*-infected mice. ^^^*P* < 0.05 vs. HP_24_-treated macrophages from *T. cruzi*-infected mice + heart explants from *T. cruzi*-infected mice. Mps, macrophages; *Tc, T. cruzi*.

To investigate whether macrophages from *T. cruzi-*infected HP_24_-treated mice participate in heart angiogenesis, we cocultured macrophages from these mice with heart explants from *T. cruzi-*infected mice. Then, we evaluated the expression of proangiogenic markers in those explants. Macrophages from *T. cruzi-*infected mice induced an increase in VEGF-A expression in heart explants, whereas macrophages from control mice were unable to modify VEGF-A expression in the same explants (Figure 5). When heart explants were cocultured with macrophages from *T. cruzi-*infected mice treated *in vitro* with HP_24_, the expression of VEGF-A, CD31 and Arg-I was further increased (Figure 5).

Moreover, the effect of HP_24_ on the expression of VEGF-A, CD31 and Arg-I in heart explants was reverted when the macrophages from *T. cruzi-*infected mice were pretreated with the PPARγ antagonist T0070907 (Figure 5).

The same pattern was observed when macrophages were cocultured with heart explants from uninfected mice although the expression levels were lower (Figure S1 in Supplementary Material). Besides, the expression of CD31, VEGF-A and Arg-I did not differ in heart explants from *T. cruzi-*infected mice cultured alone or in the presence of macrophages from uninfected control mice (Figure S2 in Supplementary Material).

### HP_24_ Treatment Reduces Inflammatory Response and Heart Fibrosis in *T. cruzi*-Infected Mice

**Figure 6:**
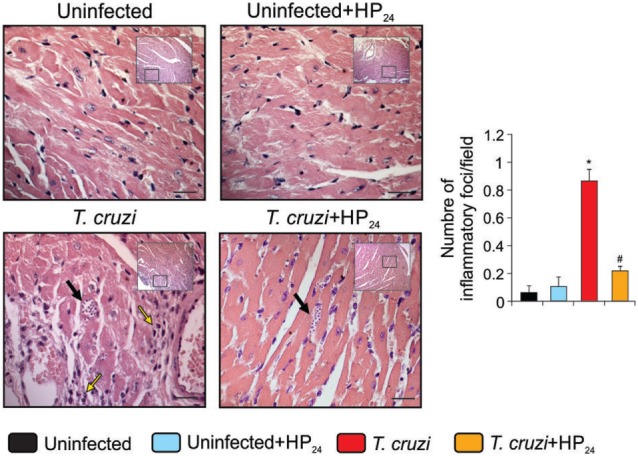
HP_24_ reduces heart inflammatory reaction in *Trypanosoma cruzi*-infected mice. Heart inflammatory reaction and parasite nests were analyzed at 10 dpi in histological sections of *T. cruzi*-infected, *T. cruzi*-infected HP_24_-treated, and uninfected mice, stained with hematoxilin–eosin (bar: 100 µm, yellow arrows, inflammatory infiltrates, black arrows, amastigote nests). Results represent the mean ± SEM of three independent experiments (five mice per group). *P < 0.05 vs. uninfected mice, ^#^*P* < 0.05 vs. *T. cruzi*-infected mice.

We investigated the effects of HP_24_ treatment on the inflammatory response in the heart of *T. cruzi-*infected mice. Mice infected with *T. cruzi* showed intense inflammatory reaction, consisting of mononuclear cell infiltrates. Treatment with HP_24_ significantly reduced heart inflammation (number of inflammatory foci/field, *T. cruzi* vs. *T. cruzi*-HP_24_, 0.84 ± 0.41 vs. 0.21 ± 0.05, *N* = 5, *P* < 0.05, Figure 6). We did not find significant differences in the number of amastigote nests *per* field between the *T. cruzi-*infected and *T. cruzi-*infected HP_24_-treated groups (Figure 6).

**Figure 7:**
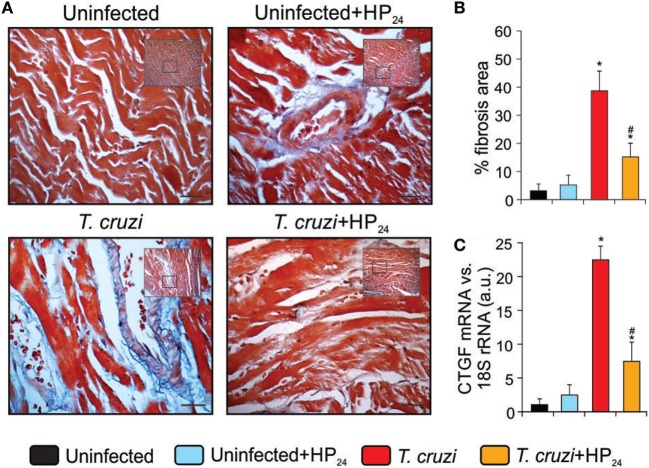
Reduction of heart fibrosis by HP_24_ in *Trypanosoma cruzi*-infected mice **(A)**.Collagen deposits in heart sections of the experimental groups were assessed using Masson’s trichrome (bar: 100 µm). **(B)** The bar graph shows the percentage of area with fibrosis. **(C)** Connective tissue growth factor (CTGF) mRNA expression was analyzed by quantitative real-time reverse-transcriptase polymerase chain reaction in heart homogenates from all experimental groups. Results were normalized against 18S rRNA. All studies were performed at 10 dpi. Results represent the mean ± SEM of three independent experiments (five mice per group). *P < 0.05 vs. uninfected mice, ^#^*P* < 0.05 vs. *T. cruzi*-infected mice.

Fibrosis was observed in heart sections of *T. cruzi*-infected mice using Masson’s trichrome staining (Figure 7A). The heart area compromised by collagen deposits was significantly reduced in *T. cruzi*-infected HP_24_-treated mice (Figure 7B). In addition, we analyzed the mRNA expression of CTGF as a profibrotic marker. RT-qPCR showed that CTGF expression levels were higher in the hearts from *T. cruzi*-infected mice than in those from uninfected mice. Interestingly, treatment with HP_24_ significantly inhibited CTGF mRNA in infected mice (Figure 7C).

### HP_24_ Treatment Increases the Expression of Proangiogenic Markers in the Heart of *T. cruzi*-Infected Mice

**Figure 8:**
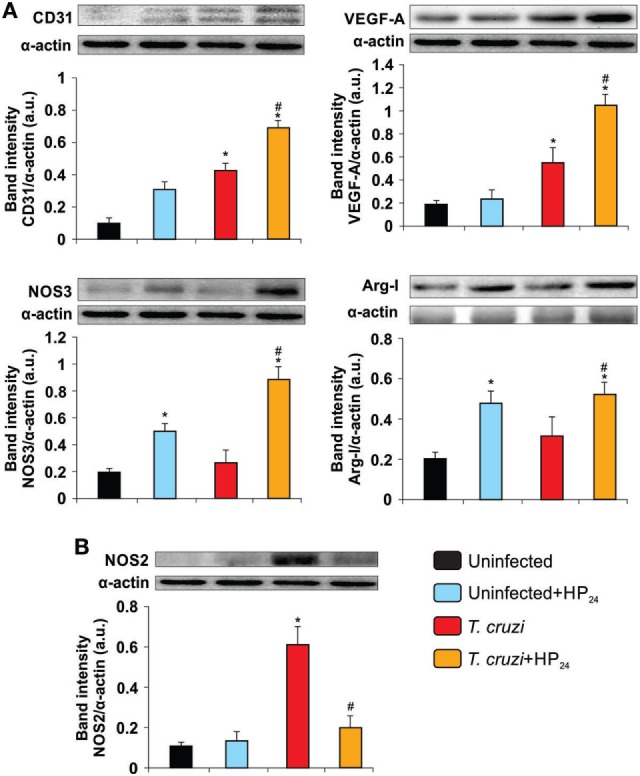
HP_24_ increases the expression of proangiogenic markers and decreases nitric oxide synthase 2 (NOS2) in the heart of mice infected with *Trypanosoma cruzi*. Expression of CD31, vascular endothelial growth factor A (VEGF-A), NOS3, Arginase I (Arg-I) **(A)**, and NOS2 **(B)** was determined in the heart by Western blot. Anti-CD31, anti-VEGF-A, anti-NOS3, anti-Arg-I, and anti-NOS2 specific antibodies were used, and protein levels were normalized against α-actin. Results show a representative experiment out of three performed. Results represent the mean band intensity ± SEM of three independent experiments (five mice per group). *P < 0.05 vs. uninfected mice, ^#^*P* < 0.05 vs. *T. cruzi*-infected mice.

To demonstrate the proangiogenic role of the HP_24_ treatment in the hearts of *T. cruzi*-infected mice, we evaluated the cardiac expression of CD31, VEGF-A, NOS3, and Arg-I. Western blot analysis showed increased expression of CD31 and VEGF-A in hearts upon infection while that of NOS3 and Arg-I remained unchanged. Treatment with HP_24_ promoted the increase of all proangiogenic markers in the heart of *T. cruzi-*infected mice. (Figure 8A). Moreover, HP_24_ treatment *in vivo* reduced the expression of NOS2 in the hearts of *T. cruzi*-infected mice in comparison with untreated *T. cruzi-*infected mice (Figure 8B).

## Discussion

In this study, we analyzed the role of the new synthetic PPARγ ligand HP_24_ (22) in cardiac damage and neovascularization, in an experimental model of Chagas disease, considering the participation of macrophages. We demonstrated that HP_24_ treatment increased the expression of proangiogenic factors, inhibited pro-inflammatory mediators, and reduced fibrosis in the heart of infected mice. Moreover, we evidenced that HP_24_ potentiates the ability of macrophages to stimulate angiogenesis in our experimental model. PPARγ and its ligands have a wide spectrum of functions, regulating metabolism, attenuating inflammation, maintaining the balance of immune cells, inhibiting apoptosis and oxidative stress, and improving endothelial function (40). Results from different experimental models have shown that PPARγ and its ligands play a critical role in the regulation of various biological processes in the cardiovascular system under pathological conditions: they attenuate cardiac fibrosis in diabetic rats (41, 42), and alleviate ischemia–reperfusion injury through the inhibition of inflammation, improve endothelial function, reduce oxidative, stress, and calcium overload in rabbits (43).

Molecular modeling used in this work predicted that the main interactions between the PPARγ and HP_24_ differ little from another synthetic agonist, RSG. Our data suggest that HP_24_ has a lower number of non-polar contacts because its volume is lower than that of RSG. Only PHE363 and CYS285 are at a suitable distance for hydrophobic interaction. In addition, the carbon 2 of the pyridine ring is at an optimal distance for a possible nucleophilic attack by the CYS285. This may be determinant for the PPARγ agonist function, since such a covalent bond is widely described for the positive regulators of this receptor (44, 45). Thus, while the interaction free energy of HP_24_ with PPARγ is higher than that of RSG, the covalent binding that might be formed between CYS285 and the pyridine ring of HP_24_ would make the binding stable.

To evaluate the implication of PPARγ in HP_24_ effects, mouse peritoneal macrophages were pretreated with the specific PPARγ antagonist T0070907 and NO production was evaluated. Reversion of the inhibitory effects of HP_24_ on the release of NO, together with the docking analysis, strongly suggests the binding of HP_24_ to PPARγ.

In the present work, we demonstrated that HP_24_ treatment increases VEGF-A expression in macrophages from *T. cruzi-*infected HP_24_-treated mice. Our results are in agreement with those of Kotlinowski et al. (16), who showed that the activation of PPARγ by rosiglitazone increases the proangiogenic potential of endothelial cells and of bone marrow-derived proangiogenic cells (16). In this regard, Biscetti et al. found that the activation of PPARγ and PPARα receptors stimulates neoangiogenesis through a VEGF-dependent mechanism (17).

We have previously demonstrated that the treatment with Wy14643 and 15dPGJ2 (PPARα and PPARγ ligands, respectively) drives peritoneal macrophages toward an M2 profile in *T. cruzi*-infected mice (30). In view of these results, we analyzed whether the treatment with HP_24_ could influence the profiling of macrophages. We showed that macrophages from *T. cruzi*-infected HP_24_-treated mice upregulated Arg-I expression and downregulated NOS2 expression, processes that strongly suggest M2 differentiation. Recently, Assunção et al. demonstrated that M2 polarization of macrophages triggered by Schistosomal-derived lipids occurs through a PPARγ-dependent mechanism (46). Interestingly, Odegaard et al. showed that the promoter region of the Arg-I gene has PPARγ response elements (47).

We previously shown that 15dPGJ2 is a potent modulator of the inflammatory processes through PPARγ-dependent and -independent pathways, in cultures of *T. cruzi*-infected neonatal cardiomyocytes and *T. cruzi*-infected mice (37, 38). Here, we determined the ability of HP_24_ to exert anti-inflammatory actions by inhibiting the expression of a pro-inflammatory enzyme as NOS2 and releasing inflammatory cytokines such as TNF-α and IL-6 in macrophages from *T. cruzi-*infected mice. In the same line of evidence, Brun et al. (22) showed that the synthetic PPARγ agonist HP_24_ improves the outcome of dextran-induced colitis, by reducing colonic myeloperoxidase activity and IL-1β levels in the gut of mice. These authors also demonstrated that HP_24_ prevents LPS-induced TNF-α and IL-8 release in the same model (22).

During acute Chagas disease, oxidative stress associated with inflammation of the heart contributes to the tissue damage triggered by *T. cruzi* infection. This condition stimulates the release of a new set of proangiogenic mediators such as VEGF, CD31, angiopoietin (Ang)-1 and Ang-2 (48). We cannot discard that macrophages participate in this angiogenic process. Regarding the latter, our group reported that peritoneal macrophages from tumor-bearing mice are able to induce a strong neovascular response (32). In this study, we showed that passive transfer of macrophages from *T. cruzi-*infected mice treated *in vitro* with HP_24_ enhances angiogenesis, as shown by the increase of VEGF-A and CD31 expression in the skin of normal syngeneic recipient mice. This effect was dependent on PPARγ signaling, since pretreatment of macrophages with the PPARγ antagonist precluded the HP_24_-induced increase of CD31 and VEGF-A in skin homogenates of syngeneic normal recipient mice.

Recently, Guedes-da-Silva et al. (49) showed that antigens from the Y strain of *T. cruzi* are able to promote inflammatory neovascularization, probably induced by angiogenic mediators produced by macrophages (49). Furthermore, Shrestha et al. (50) demonstrated that *T. cruzi* infection increases the inflammatory and angiogenic mediators in the heart of infected mice (50). Taking into account that the evolution of Chagas disease is linked to microvascular lesions, including obstruction due to thrombosis, perivascular inflammation, and lesions in the coronary arterioles (33), we believe that the increased neovascularization observed during *T. cruzi* infection may act as a delaying factor of the physiopathological mechanisms leading to the cardiac symptoms during the course of Chagas disease.

Peritoneal macrophages from *T. cruzi-*infected mice treated *in vitro* with HP_24_ induced higher expression of VEGF-A, CD31, and Arg-I than macrophages from *T. cruzi-*infected mice in heart explants. As observed in the skin model, these findings suggest that HP_24_ plays a role in inducing macrophages from infected mice to promote cardiac angiogenesis. In fact, similarly to what was found in the case of passive transfer to the skin, the effect of HP_24_ on the expression of VEGF-A, CD31, and Arg-I depended on PPARγ, since pretreatment of macrophages with the PPARγ antagonist T0070907 significantly reduced the expression of proangiogenic markers in heart explant homogenates. Ashoff et al. demonstrated that treatment of diabetic rats with pioglitazone, a synthetic PPARγ agonist, increases the density of capillaries in heart and skeletal muscle (51). Besides, de la Torre et al. (33) demonstrated that LPS-treated macrophages from septic mice show increased CD31 and VEGF-A expression in the heart (33).

However, some studies have shown that PPARγ has antiangiogenic activity. Pigment epithelial-derived factor and its functional peptides inhibit angiogenesis by means of upregulation of PPARγ in a rat model of acute myocardial infarction and in an *in vitro* model of myocardial angiogenesis (52). Funovics et al. (53) and Kim et al. (54) have shown that PPARγ activation results in reduced expression of VEGF-R2 and VEGF-R1 in endothelial cells (53, 54). These seemingly contradictory findings highlight the need for additional studies to determine not only the mechanisms through which different PPAR ligands promote angiogenesis but also the physiopathological significance of the increase of the proangiogenic markers and mediators in the case of Chagas disease.

The myocardial infection caused by *T. cruzi* elicits an intense inflammatory response that may act as a “double-edged” sword. Although necessary for the control of parasite proliferation, inflammation results in tissue damage, leading to myocardial fibrosis and remodeling. CTGF, a fibrogenic cytokine, has hypertrophic effects, modulating the myocardial phenotype (55). In this regard, Koitabashi et al. (56) demonstrated that a disproportionate increase in CTGF expression in cardiac myocytes plays a central role in the induction of excessive myocardial fibrosis and diastolic heart failure (56). In this study, we showed that treatment of *T. cruzi*-infected mice with HP_24_ significantly reduces inflammatory response and heart fibrosis, and inhibits CTGF expression. We have recently reported that 15dPGJ2, a natural PPARγ ligand, is able to reduce the inflammatory response, fibrosis, and enzyme markers of liver damage in *T. cruzi*-infected mice (34). It has been shown that activation of PPARγ by pioglitazone can attenuate cardiac fibrosis and partly ameliorate cardiac remodeling and function by suppressing activity of RAS, in a rat model of diabetes (42). Here, we demonstrated that, besides reducing heart fibrosis, HP_24_ treatment induces the expression of proangiogenic markers (VEGF-A, CD31, Arg-I, and NOS3) and reduces that of NOS2, in the heart of *T. cruzi*-infected mice.

In conclusion, taking into account that during *T. cruzi* infection microvascular lesions contribute to ischemia and necrosis, the early treatment with drugs that reduce inflammation and fibrosis, and simultaneously increase microvascular development, might be useful as a coadjuvant of the antiparasitic treatment to delay, reverse, or preclude the onset of heart damage during the course of Chagas disease.

## Ethics Statement

All the procedures were approved by the Institutional Committee for the Care and Use of Laboratory Animals (CICUAL, Facultad de Medicina, Universidad de Buenos Aires, CD No. 2271/2014) and are in accordance with the guidelines of the Argentinean National Administration of Medicines, Food and Medical Technology (ANMAT), Argentinean National Service of Sanity and Agrifoods Quality (SENASA) and also based on the US NIH Guide for the Care and Use of Laboratory Animals.

## Author Contributions

NG, MS, and FP designed experiment; FP, NG, and GM contributed to the writing of the manuscript; FP, GD, GM, ÁC, and MR did experiments; FP, NG, MS, and GM analyzed data; MF and DC provided the 3-hydroxy-4-pyridinecarboxylic acid derivative (HP_24_); NG, MS, GM, and FP contributed to final approval of the version to be published.

## Conflict of Interest Statement

The authors declare that the research was conducted in the absence of any commercial or financial relationships that could be construed as a potential conflict of interest.
