# Supplementary material for: Treatment with a New Peroxisome Proliferator-Activated Receptor Gamma Agonist, Pyridinecarboxylic Acid Derivative, Increases Angiogenesis and Reduces Inflammatory Mediators in the Heart of Trypanosoma cruzi-Infected Mice
Source: Front Immunol. 2017 Dec 11;8:1738. doi: 10.3389/fimmu.2017.01738 (PMC5732351; doi:10.3389/fimmu.2017.01738)
Supplement: Supplementary file 1 [file Data_Sheet_1.doc]

**SUPPLEMENTARY FIGURES**

**
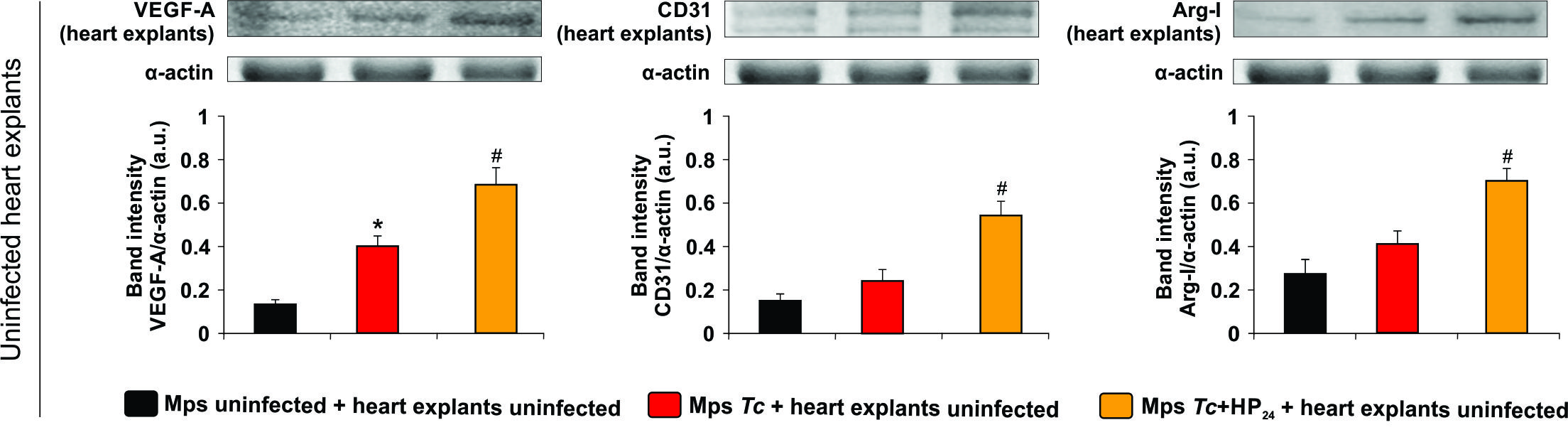
**

**Supplementary Figure S1. Effect of peritoneal macrophages from *T. cruzi*-infected HP_24_-treated mice on the expression of proangiogenic proteins in heart explants from uninfected mice.** Peritoneal macrophages were obtained from mice uninfected, *T. cruzi*-infected and *T. cruzi*-infected HP_24_-treated mice at 10 dpi and co-cultured with heart explants (100 mg/sample) from uninfected mice. After 48 h, heart explants were collected and homogenized. VEGF-A, CD31 and Arginase I (Arg-I) expressions were determined by Western blot with specific antibodies. Protein levels were normalized against α-actin. Results represent the mean ± SEM of three independent experiments. * P < 0.05 vs. uninfected macrophages + uninfected heart explants # P < 0.05 vs. macrophages from *T. cruzi*-infected mice + uninfected heart explants. Mps: macrophages; *Tc*: *T. cruzi.*


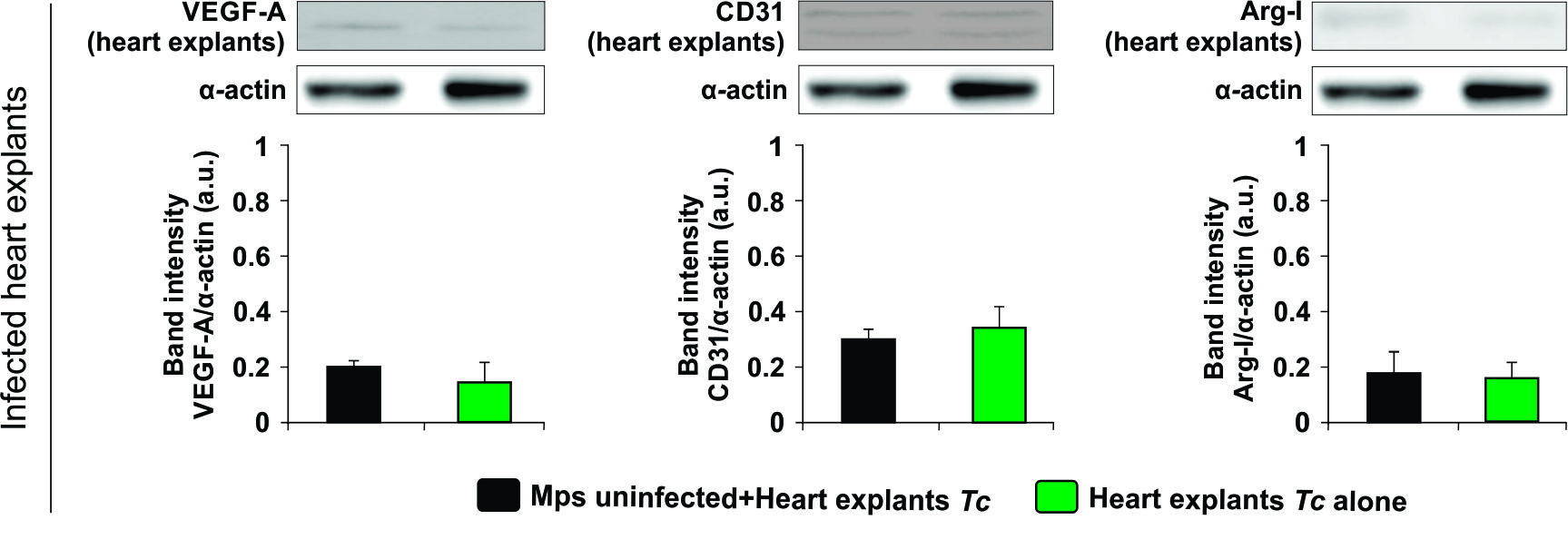


**Supplementary Figure S2. Expression of proangiogenic proteins in *T. cruzi*-infected heart explants alone or *T. cruzi*-infected heart explants co-cultured with peritoneal macrophages from uninfected mice.** VEGF-A, CD31 and Arginase I (Arg-I) expressions were determined by Western blot with specific antibodies in heart explants from *T. cruzi*-infected mice alone (100 mg/sample) or *T. cruzi*-infected heart explants co-cultured with peritoneal macrophages obtained from uninfected mice. After 48 h, heart explants were collected and homogenized. Protein levels were normalized against α-actin. Results represent the mean ± SEM of three independent experiments. Mps: macrophages; *Tc*: *T. cruzi.*
